# Growth Performance and Nutrient Composition of Mealworms (*Tenebrio Molitor*) Fed on Fresh Plant Materials-Supplemented Diets

**DOI:** 10.3390/foods9020151

**Published:** 2020-02-05

**Authors:** Changqi Liu, Jasmin Masri, Violet Perez, Cassandra Maya, Jing Zhao

**Affiliations:** 1 School of Exercise and Nutritional Sciences, San Diego State University, San Diego, CA 92182, USA; changqi.liu@sdsu.edu (C.L.); cmaya6248@sdsu.edu (C.M.); 2 School of Kinesiology and Nutritional Science, Rongxiang Xu College of Health and Human Services, California State University, Los Angeles, CA 91803, USA; j.masri13@gmail.com (J.M.); vperez59@calstatela.edu (V.P.)

**Keywords:** mealworm, feed supplementation, growth performance, nutrient composition, antioxidant activity

## Abstract

Mealworms (*Tenebrio molitor*) have a great potential to serve as a sustainable food source for humans due to their favorable nutrient profile and low environmental impact. Feed formulation and optimization are important for mealworm production. The objective of this study was to evaluate the effects of fresh plant materials-supplemented diets on the growth performance and nutritional value of mealworms. Mealworm larvae were grown on wheat bran or wheat bran enriched with carrot, orange, or red cabbage for four weeks. Larval and pupal survival, growth rate, pupating rate, duration of pupal stage, proximate composition, reducing power, metal chelating activity, and radical scavenging activity of the mealworms were analyzed. Dietary supplementation with fresh plant materials did not result in significant changes in mealworm survival, development, proximate composition, or antioxidant activities. However, mealworm larvae fed on carrot-, orange-, and red cabbage-supplemented diets had improved growth rates, and were 40%–46% heavier in week four than those fed on wheat bran only, indicating the supplementation resulted in an increased production efficiency of mealworm larvae. Our findings may help optimize the diet formulation for mealworm mass production.

## 1. Introduction

Entomophagy, the human consumption of insects, is practiced in many regions including parts of Africa, Asia, and Latin America [1,2]. Although Western acceptance of entomophagy remains low [3], the utilization of edible insects as human food has received increasing attention in recent years, particularly after the Food and Agriculture Organization (FAO) of the United Nations recommended using insects as a sustainable alternative to the traditional livestock [4]. Insects are rich in high quality proteins, polyunsaturated fatty acids, dietary fibers, and a variety of micronutrients [5]. In addition to the nutritional benefits, insects have a high feed conversion rate, low environmental footprints, and are significantly less land-dependent than livestock production [6]. Globally, over 1500 edible insect species are consumed [7], many of which have the potential of becoming part of the Western diet. However, one must exercise caution when selecting nonnative insects for farming, as they may be invasive and detrimental to the environment. For example, palm weevils (*Rhynchophorus* spp.) are insects widely consumed in Africa, South America, Southeast Asia, and New Guinea [7], but are also devastating pests of palm trees in California [8]. 

*Tenebrio molitor* L. (Coleoptera: Tenebrionidae), commonly known as mealworm, has been commercially produced in the US for over 70 years [9] and is a favorable candidate for insect rearing due to its high protein content, well-balanced amino acid profile [5], potential health benefits [10,11,12], efficient feed conversion rate [13], low greenhouse gas emissions [14], low water footprint [15], reduced land usage [9], ability to live on organic by-products [16], and available mass production technology [9]. Although mealworms can be reared exclusively on wheat bran, their diets are often enriched with additional organic matters such as potato, carrot, and cabbage to provide important nutrients [9]. Effects of dietary supplementation on mealworm survival, growth, development, fecundity, feed conversion, and nutrient composition have been reported in previous research works [13,16,17,18,19,20,21]. However, few studies have focused on antioxidant-rich supplements. Insects have a tracheal respiratory system that directly delivers oxygen to tissues and results in high levels of reactive oxygen species. Therefore, insects are prone to oxidative stress that can negatively impact their growth, development, survival, and fecundity [22]. The objective of this study was to investigate whether dietary supplementation of carrot, orange, and red cabbage as a source of antioxidants can improve growth performance, nutrient composition, and antioxidant activity of mealworm larvae.

## 2. Materials and Methods

### 2.1. Materials

Mealworm larvae and wheat bran were purchased from Mulberry Farms (Fallbrook, CA, USA). Shredded carrots, oranges, and red cabbages were purchased from a local grocery store (Los Angeles, CA, USA) and refrigerated (4 °C) until use. Reagent-grade chemicals were purchased from Fisher Scientific (Hampton, NH, USA).

### 2.2. Feeding

Mealworm larvae were divided into four different dietary groups: (1) 50 g of wheat bran only (control), (2) 50 g of wheat bran supplemented with 20 g of fresh carrot per day, (3) 50 g of wheat bran supplemented with 20 g of fresh orange per day, and (4) 50 g of wheat bran supplemented with 20 g of fresh red cabbage per day. Each group consisted of 60 g of mealworm larvae (approximately 670 mealworms). Mealworms were reared with an ad libitum supply of feed for four weeks. The feed was replaced weekly to rid the environment of insect waste and uneaten feed.

### 2.3. Growth Performance

Mealworm pupae and dead larvae were removed daily from each group, and the insect numbers were recorded. The separated pupae were observed daily for eclosed beetles. The average weight of the mealworm larvae was recorded each week on 20–30 randomly selected worms. The average weight of the pupae and beetles and the duration of pupal stage were recorded.

### 2.4. Sample Preparation

Mealworm larvae were sampled each week for analysis. Mealworms were fasted for 24 h and then euthanized by freezing at −40 °C for 10 min. For proximate analyses, mealworms were finely ground using a blender. For antioxidant activity assays, one gram of mealworm larvae was homogenized using a Polytron PT 3000 homogenizer (Kinematica AG, Luzern, Switzerland) in 10 mL of 70% (v/v) ethanol aqueous solution until no large pieces were visible (approximately 20 s). The homogenized samples were centrifuged using a Biofuge Stratos centrifuge (Kendro Laboratory Products, Asheville, NC, USA) at 5000× *g* for 10 min at 4 °C. The supernatants were collected for the analyses.

### 2.5. Proximate Analysis

The proximate composition of mealworm larvae was analyzed according to the AOAC Official Methods [23]. Moisture content was measured by drying the samples in a convection oven (Freas, Marietta, OH, USA) at 125 °C for 90 min. Total crude fat was extracted in petroleum ether using an ST243 Soxtec solvent extraction system (Foss, Hilleroed, Denmark). Nitrogen content was determined using a rapid N exceed nitrogen analyzer (Elementar, Hesse, Germany). A nitrogen-to-protein conversion factor of 4.76 was used for protein quantification [24]. Ash content was measured by incinerating the samples in an Isotemp Programmable muffle furnace (Fisher Scientific, Hampton, NH, USA) at 550 °C for 48 h. Total carbohydrate was calculated by the difference.

### 2.6. Ferric Reducing Power

The ferric reducing power of the mealworm 70% ethanol extract was analyzed according to Oyaizu [25] with modifications. In microcentrifuge tubes, 15 µL of the sample extract was mixed with 125 µL of 0.2 M sodium phosphate buffer (pH 6.6) and 125 µL of 1% (w/v) potassium ferricyanide solution, then vortexed and incubated for 20 min at room temperature. In a 96-well plate, 125 µL of the mixture was pipetted into each well followed by addition of 125 µL of deionized water and 25 µL of 1% (w/v) ferric chloride solution. Color was developed for 5 min at room temperature and absorbance was read at 700 nm using a Synergy H1 microplate reader (BioTek, Winooski, VT, USA). Ferric reducing power was calculated using a 0.28–2.84 mM ascorbic acid standard curve (*r* = 0.99). 

### 2.7. Ferrous Chelating Activity

Ferrous chelating activity was determined according to Carter [26] with modifications [27]. In a 96-well microplate, 20 µL of the sample extract was mixed with 125 µL of deionized water and 10 µL of 2 mM freshly prepared ferric chloride solution. The plate was set aside for 5 min at room temperature before adding 45 µL of 5 mM Ferrozine into each well. The contents of each well were gently mixed via pipetting and set aside for 10 min to allow for color development. The absorbance was read at 562 nm using a microplate reader and ferrous chelating activity was calculated using a 0.34–1.37 mM ethylenediaminetetraacetic acid (EDTA) standard curve (*r* = 0.99). 

### 2.8. ABTS Radical Scavenging Activity

Seven millimolar 2,2’-azino-di-(3-ethylbenzthiazoline sulfonic acid) (ABTS) was mixed with 2.45 mM potassium persulfate to prepare the ABTS^•+^ stock solution. The reagent was kept in the dark for 12–16 h at room temperature. Before use, the ABTS^•+^ stock solution was diluted in 10 mM sodium phosphate buffer (pH 7.2) to an absorbance of ~0.7 at 734 nm. In a cuvette, 20 µL of the sample extract was mixed with 1.980 mL of the diluted ABTS^•+^ solution and allowed to stand for 10 min. Absorbance was read at 734 nm using a GENESYS 10S spectrophotometer (Thermo Scientific, Waltham, MA, USA) and ABTS radical scavenging activity was calculated using a 0.40–2.40 mM Trolox standard curve (*r* = 0.99) [28]. 

### 2.9. Statistical Analysis

All experiments were repeated at least twice with triplicate measurements for each replication. Data were analyzed using R (version 3.6.0) with emmeans and multcomp packages. One-way analysis of variance (ANOVA) and Tukey’s honestly significant difference (HSD) test at α = 0.05 were used for the overall analysis of variance and mean separation, respectively. 

## 3. Results and Discussion

### 3.1. Growth Performance

**Figure 1:**
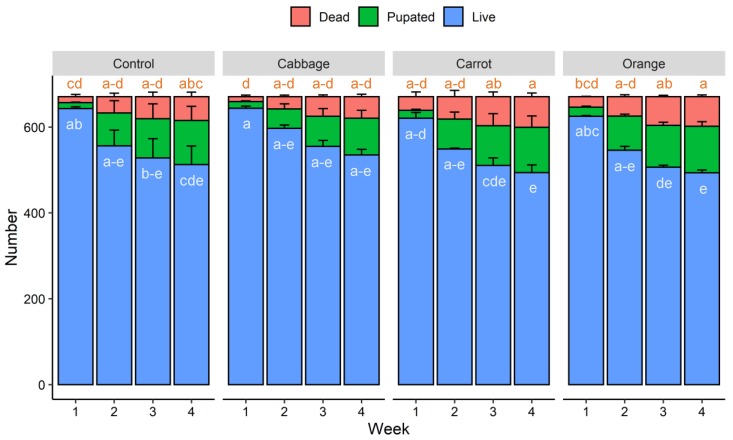
Number of dead, pupated, and live mealworm larvae fed on different diets over four weeks. Numbers of dead or live mealworm larvae sharing no common letters differ significantly (*p* < 0.05). No significant difference was found in the numbers of pupated mealworm larvae.

Over four weeks, the larval survival rates of mealworms fed on the control diet (wheat bran only) and diets supplemented with red cabbage, carrot, and orange were 91.7%, 92.5%, 89.3%, and 89.7%, respectively, and pupating rates of the larvae were 15.4%, 12.7%, 15.7%, and 16.2%, respectively (Figure 1). Within each week, no significant difference (*p* > 0.05) in the number of live, dead, and pupated mealworm larvae was detected between diet groups.

**Figure 2:**
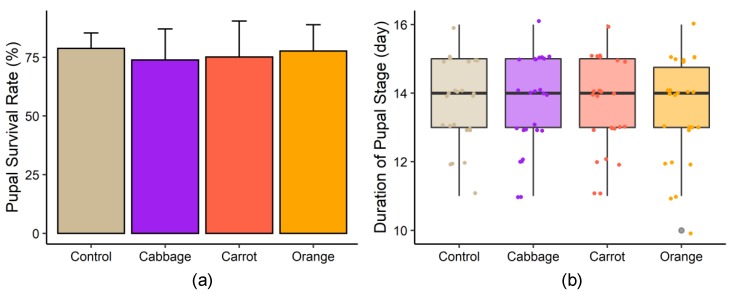
(**a**) Pupal survival rate and (**b**) pupa-to-beetle development time of mealworms fed on different diets.

The survival rates of pupated mealworms fed on the control diet and red cabbage-, carrot-, and orange-enriched diets were 78.8%, 73.9%, 75.1%, and 77.7%, respectively (Figure 2a). The average time required for pupal development and eclosion to beetles was 14 days in all experimental groups (Figure 2b). No significant difference was found in either pupal survival rate or duration of pupal stage between diet groups.

**Figure 3:**
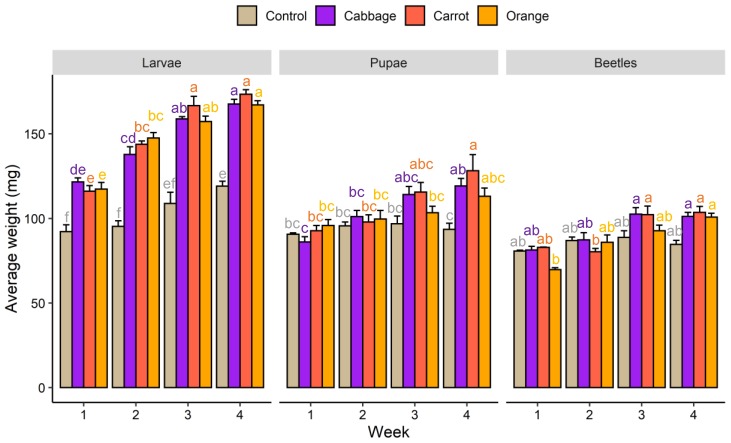
Average weight of mealworm larvae, pupae, and beetles fed on different diets. Means within each metamorphosis stage sharing no common letters differ significantly (*p* < 0.05).

Average weight of mealworm larvae fed on wheat bran increased 29.3% over four weeks (Figure 3). Dietary supplementation with red cabbage, carrot, and orange improved the growth rates to 37.9%, 49.3%, and 42.5%, respectively. In week four, mealworm larvae fed on fresh plant materials-supplemented diets were 40%–46% heavier (*p* < 0.05) than those fed on wheat bran only. Whether the diet was supplemented with red cabbage, carrot, or orange did not result in a significant difference in the average weight of mealworm larvae. The weight increment as a result of diet enrichment diminished after pupation and eclosion. The only significant differences observed were higher pupae weights in the red cabbage and carrot groups as compared to the control group in week four (*p* < 0.05).

Mealworms can obtain all required nutrients for growth, development, and reproduction from wheat bran [9]. Although feeding experiments have shown improvements in larval survival and development time when additional ingredients were provided, most of the studies have focused on supplementing and balancing macronutrients such as protein, starch, and fat [13,16,17,18,19,20]. The provision of carrot, orange, and red cabbage did not alter the survival or development time of mealworms in our study. This was likely due to the low concentrations of macronutrients (0.93%–1.43% protein, 0.12%–0.24% lipid, and 7.37%–11.75% carbohydrate) in the supplemented fresh plant materials [29]. Our observations were in agreement with a previous study that reported lack of improvement in house cricket (*Acheta domesticus* L.) growth performance after carrot supplementation [30]. However, another study reported that carrot consumption went up in mealworms fed on a diet of poor nutritional quality as compared to those fed on high quality diets, suggesting that carrot was utilized as a source of nutrients to compensate for poor diet quality [16].

The weight of mealworm larvae increased significantly when carrot, orange, and red cabbage were provided. This was likely due to the high moisture content of these plant materials (86.75%–90.39%) [29]. Mealworm larvae are highly drought tolerant and can actively absorb water from air [31]. Yet, they grow faster when water or high moisture foods are provided [9]. This is partly due to the reduced energy requirement for active water vapor absorption [31]. Moreover, the fresh plant materials may provide important micronutrients and bioactive phytochemicals. Orange and red cabbage are rich in ascorbic acid (53.2 mg/100 g and 57 mg/100g, respectively) [29], a vitamin needed for growth, molting, and fertility of many insects [32]. Although ascorbic acid is not an essential nutrient for mealworms [32,33], it may reduce oxidative stress that is known to retard insect growth [22]. Carrot is a good source of carotenoids such as β-carotene (8.29 mg/100 g), α-carotene (3.48 mg/100 g), lutein and zeaxanthin (256 μg/100 g) [29]. These carotenoids are potent antioxidants and can stimulate immune system of invertebrates [34]. However, supplementation with carotenoids may produce mixed results. Lifetime dietary supplementation with 0.1 mg astaxanthin/larva/week has resulted in reduced immunity, growth rate, and survival of mealworm larvae [35]. This was likely due to the interactions between astaxanthin and nitric oxide, a compound involved in both cellular and humoral immunity of insects. Astaxanthin inhibits nitric oxide synthase and scavenges circulating nitric oxide and consequently suppresses the insect immune responses [34]. It is noteworthy that red cabbage is a source of allelochemicals such as glucosinolates and flavonoids (e.g., anthocyanins). Glucosinolates are known to inhibit respiration of several insects [36] including mealworms [37]. Flavonoids can inhibit respiration and growth performance of some insects [36]; however, such detrimental effects have not been reported in mealworms. No deterrent or harmful effects of red cabbage on mealworms were observed in our study.

### 3.2. Proximate Composition

**Figure 4:**
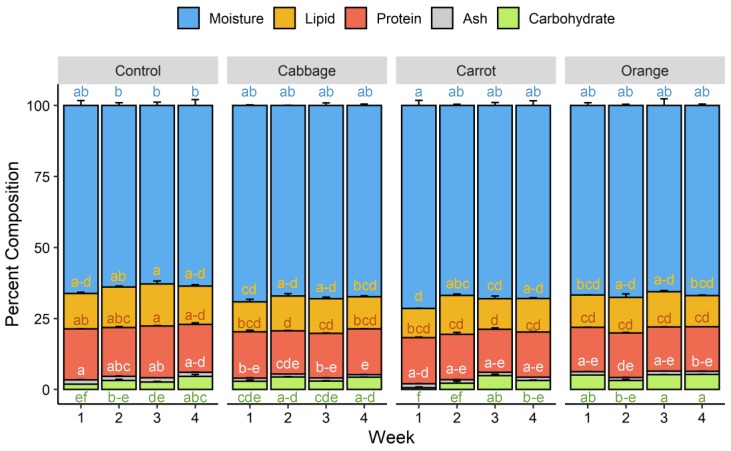
Proximate composition of mealworm larvae fed on different diets over four weeks. Means of each composition sharing no common letters differ significantly (*p* < 0.05).

The larval stage of mealworm is the most favorable for efficient production and safe consumption [38]. Therefore, proximate composition was only determined for mealworm larvae and not for the pupae and beetles. Mealworm larvae fed on the control diet had a proximate composition of 64.1% moisture, 13.8% lipid, 17.6% protein, 1.5% ash, and 3.1% carbohydrate, or on a dry weight basis, 38.3% lipid, 49.1% protein, 4.1% ash, and 8.5% carbohydrate, which was comparable to the values reported in the literature [5]. Due to the presence of non-protein nitrogen in mealworms, a nitrogen-to-protein conversion factor of 4.76 was used for protein quantification [24]. Diet supplementation with fresh plant materials did not result in appreciable changes in proximate composition of mealworms over four weeks (Figure 4). This result was expected as the supplements are not a significant source of major nutrients. Moreover, the nutrient intake of insects is well regulated. When given the opportunity, insects feed selectively to reach their nutrient target. When the diet is restricted as in our experiment, insects employ postingestive regulations to balance the nutrient intake [39]. These mechanisms explain the relatively stable protein content in mealworms fed on diets that differed 2–3 fold in protein content [16]. The excess proteins ingested were eliminated as uric acid and possibly ammonia [16,39].

### 3.3. Antioxidant Activity

While insects largely rely on endogenous antioxidant enzyme systems to balance the prooxidant-antioxidant homeostasis, dietary supplies of antioxidant can complement this process [22]. The supplemented carrot, orange, and red cabbage are rich in antioxidants such as β-carotene (carrot), ascorbic acid (orange and red cabbage), and anthocyanins (red cabbage) [29,40]. Beta-carotene inhibits lipid peroxidation by quenching singlet oxygens and scavenging peroxyl radicals [41]. Ascorbic acid neutralizes reactive oxygen species and plays pivotal roles in auxiliary antioxidant enzyme systems of insects [22,42]. Anthocyanins scavenge free radicals and inhibit the formation of highly reactive hydroxyl radicals by chelation with ferrous ions [43]. The observed improvement in larvae growth rate in the carrot, orange, and red cabbage groups may be partly attributed to the supplemented antioxidants. Antioxidant activities of the mealworm larvae fed on different diets were tested at the end of each week. Mealworms in all diet groups exhibited little variation in ferric reducing power (7.99–13.10 μmol ascorbic acid equivalent/g dry mass), ferrous chelating activity (34.74–49.96 μmol EDTA equivalent/g dry mass), and ABTS radical scavenging activity (40.40–62.17 μmol Trolox equivalent/g dry mass) over four weeks (Table 1). The ABTS radical scavenging activity was comparable to reported Trolox equivalent antioxidant capacity of mealworm aqueous extract [44]. No clear trend of antioxidant bioaccumulation as a result of dietary supplementation was observed. Although insects are known to accumulate carotenoids [45], such an effect was not noticeable in the mealworm larvae fed on carrot-supplemented diet under the tested conditions.

## 4. Conclusions

In conclusion, dietary supplementation with fresh carrot, orange, and red cabbage for four weeks improved the growth rate of mealworm larvae without changing their survival rate, development time, or proximate composition. No accumulation of antioxidant activities in mealworm larvae fed on antioxidant-rich fresh plant materials was observed under the tested conditions. Our results suggested that dietary supplementation with fresh plant materials may accelerate the growth of mealworm larvae and shorten the time required to reach a desired weight for harvesting, and thereby improve the efficiency for mass production of mealworms.

## Figures and Tables

**Table 1:** Antioxidant activity of mealworm larvae fed on different diets over four weeks.

Diet	Week	Ferric Reducing Power (μmol Ascorbic Acid Equivalent/g Dry Mass)	Ferrous Chelating Activity (μmol EDTA Equivalent/g Dry Mass)	ABTS Radical Scavenging Activity (μmol Trolox Equivalent/g Dry Mass)
Control	1	13.10 ± 0.71 ^a^	36.41 ± 0.06 ^d,e^	55.05 ± 1.10 ^a,b^
	2	11.86 ± 0.19 ^a,b,c^	41.18 ± 0.47 ^c^	46.99 ± 0.88 ^a,b^
	3	12.97 ± 0.81 ^a,b^	36.47 ± 0.22 ^d,e^	45.06 ± 2.50 ^a,b^
	4	11.66 ± 0.98 ^a,b,c^	36.64 ± 0.19 ^d,e^	58.80 ± 6.41 ^a,b^
Cabbage	1	8.50 ± 1.20 ^c^	37.77 ± 0.08 ^d^	55.20 ± 4.53 ^a,b^
	2	11.94 ± 1.23 ^a,b,c^	43.77 ± 0.23 ^b^	49.81 ± 1.65 ^a,b^
	3	10.52 ± 0.72 ^a,b,c^	38.35 ± 0.19 ^d^	46.91 ± 3.51 ^a,b^
	4	9.82 ± 0.40 ^a,b,c^	37.89 ± 0.31 ^d^	61.77 ± 7.06 ^a^
Carrot	1	10.90 ± 0.58 ^a,b,c^	43.74 ± 0.11 ^b^	54.22 ± 3.50 ^a,b^
	2	8.86 ± 0.85 ^b,c^	49.96 ± 1.22 ^a^	46.42 ± 3.87 ^a,b^
	3	11.98 ± 0.61 ^a,b,c^	43.93 ± 0.01 ^b^	47.39 ± 1.10 ^a,b^
	4	10.55 ± 0.79 ^a,b,c^	43.98 ± 0.14 ^b^	62.17 ± 1.91 ^a^
Orange	1	8.39 ± 0.83 ^c^	34.77 ± 0.27 ^e^	53.97 ± 5.75 ^a,b^
	2	7.99 ± 1.09 ^c^	40.42 ± 0.43 ^c^	48.65 ± 3.76 ^a,b^
	3	9.63 ± 1.01 ^a,b,c^	34.74 ± 0.21 ^e^	40.40 ± 1.56 ^b^
	4	10.80 ± 0.78 ^a,b,c^	35.10 ± 0.08 ^e^	58.34 ± 7.77 ^a,b^

Values are expressed as mean ± standard error. Means in the same column sharing no common letters are significantly different (*p* < 0.05). EDTA: ethylenediaminetetraacetic acid. ABTS: 2,2’-azino-di-(3-ethylbenzthiazoline sulfonic acid).
